# Association of spectroscopically determined leaf nutrition related traits and breeding selection in *Sassafras tzumu*

**DOI:** 10.1186/s13007-021-00734-5

**Published:** 2021-03-31

**Authors:** Jun Liu, Yang Sun, Wenjian Liu, Zifeng Tan, Jingmin Jiang, Yanjie Li

**Affiliations:** 1 Research Institute of Subtropical Forestry, Chinese Academy of Forestry, Fuyang, 311400 Zhejiang China; 2 College of Forestry, Nanjing Forestry University, Nanjing, People’s Republic of China

**Keywords:** Spectroscopy, Anthocyanins (ANTH), Flavonoids (FLAV), Nitrogen balance index (NBI), breeding selection

## Abstract

**Background:**

Plant traits related to nutrition have an influential role in tree growth, tree production and nutrient cycling. Therefore, the breeding program should consider the genetics of the traits. However, the measurement methods could seriously affect the progress of breeding selection program. In this study, we tested the ability of spectroscopy to quantify the specific leaf nutrition traits including anthocyanins (ANTH), flavonoids (FLAV) and nitrogen balance index (NBI), and estimated the genetic variation of these leaf traits based on the spectroscopic predicted data. Fresh leaves of *Sassafras tzumu* were selected for spectral collection and ANTH, FLAV and NBI concentrations measurement by standard analytical methods. Partial least squares regression (PLSR), five spectra pre-processing methods, and four variable selection algorisms were conducted for the optimal model selection. Each trait model was simulated 200 times for error estimation.

**Results:**

The standard normal variate (SNV) to the ANTH model and 1st derivatives to the FLAV and NBI models, combined with significant Multivariate Correlation (sMC) algorithm variable selection are finally regarded as the best performance models. The ANTH model produced the highest accuracy of prediction with a mean R^2^ of 0.72 and mean RMSE of 0.10%, followed by FLAV and NBI model (mean R^2^ of 0.58, mean RMSE of 0.11% and mean R^2^ of 0.44, mean RMSE of 0.04%). High heritability was found for ANTH, FLAV and NBI with *h*^*2*^ of 0.78, 0.58 and 0.61 respectively. It shows that it is beneficial and possible for breeding selection to the improvement of leaf nutrition traits.

**Conclusions:**

Spectroscopy can successfully characterize the leaf nutrition traits in living tree leaves and the ability to simultaneous multiple plant traits provides a promising and high-throughput tool for the quick analysis of large size samples and serves for genetic breeding program.

## Background

Nitrogen (N) is one of the most essential nutrients in plant growth, which is needed for the improvement of grain yield and quality [10]. Excessive N fertilizer application creates severe environmental problems, while inadequate N availability limits productivity. Hence, precise N application in plant is an important goal [74]. The N status of the plant should be precisely measured during growth to guide precise fertilization [63]. N is the most common limiting factor for the individual, natural and artificial ecosystems growth of the plant. Plants require N to maintain for growth mainly through external and internal sources, including soil organic matter, fertilizers, atmospheric deposition and stored N by plant themselves [52]. Plants, such as boreal species, store N seasonally through the process of internal cycling and it is a major source of N supplement for tree growth especially when the external availability of N is limited [54, 59]. Trees store N as proteins mainly in their perennial wood and bark tissues in summer and winter. In addition, other parts of foliage trees, like roots and leaves, also store N which provides nutrition for young roots and needles development. Tree N remobilisation often occurs during the growth season. The stored N mainly determines the amount of N remobilised and plays an important role for the tree seasonal growth [2, 11, 82]. The dynamics and mobilization of N stored in trees have been widely studied [15, 45]. The variation of plant species, genotype, soil and environment leads to the diversity of leaf nitrogen content [20, 73]. It is reported that the chlorophyll content has a strong positive correlation with N content which is an estimative index for N status in leaf [87]. Chlorophyll content is measured as a proxy for leaf N status [19] and non-destructive, spectroscopic, chlorophyll meters have been available for decades [19, 31, 33, 48, 56, 57]. In addition to chlorophyll, the content of flavonoids (FLAV), one of the main polyphenolic components in plant, is also correlated with the N status of the leaf [80]. Evidence shows that the rise of N fertilization will lead to flavonoid content decreasing and chlorophyll content increasing [60]. Another N status index, N balance index (NBI),which is the ratio of chlorophyll to flavonoid, is verified that has a better and more reliable correlation with leaf N concentration than chlorophyll content alone [80].

Anthocyanins (ANTH) are a group of water soluble flavonoid pigments that occur in all plant tissues. Anthocyanins are mostly related to a wide range of plant colour but often appear as red [13]. In addition, unfavourable conditions will transiently have an impact on anthocyanins accumulation in both juvenile and senescent observable plant leaves [25, 58, 81]. Thus, Anthocyanins are taken as an indicator of plant leaf senescence and stresses [44, 62].

However, research on plant growth and the variation of N storage and remobilization have typically required labour intensive methods to measure the N concentration and index properties (NBI, ANTH, and FLAV), such as atomic absorption spectrometry [8, 32], chromatography [68] and so on. These analytical methods will limit the breeding selection of tree growth with a large number of samples.

Alternatively, Near-infrared spectroscopy (NIRS) is a rapid, high-throughput technique that has been used for chemical components analysis in many fields. NIRS is a promising and reliable method that can be used for the assessment of a large number of samples [23, 27, 40, 46, 67]. NIRS relies on the absorption of light at specific wavelengths due to the vibration, stretching and bending of molecular bonds, including C–H, N–H and O–H bonds [6, 72].

Multivariate methods such as partial least squares regression (PLSR) [86] has been used to create a prediction model between NIR spectra and the independent chemical measurements. PLSR holds the advantages of producing reliable coefficients, reducing the bias and estimated error, and consuming fewer PLSR components, all of which make it one of the most popular methods for chemometric analyses [1, 7]. The model will then be applied to unknown samples by their spectra data for independent chemical prediction. Our recent research shows that leaf chlorophyll content and colour parameters are predictable on fresh leaf samples with field near infrared spectrophotometry [41]. The total FLAV and ANTH concentration also have been predicted by a general calibration model in *Ginkgo biloba* leaf and four Indonesian herbal plant species, including *Syzygium oleana*, *Piper betle*, *Jasminum* and *Graptophyllum pictum* with NIR reflectance spectroscopy. NIR is a promising tool for tree breeding selection programs due to its robustness and capacity to screen large numbers of samples [26, 41].

The robustness and reliability of model accuracy are largely determined by the spectra quality and feature selection. The combinations vibrations information and noise of the raw NIR spectra [89] will result in overlapping and difficulty to directly distinguish the target plant properties [34]. Spectra pre-processing methods, can efficiently reduce the overlapping and noise influence, such as SNV [3], 1st and 2nd derivatives and so on [36, 61]. To yield a robust and reliable model and avoid the influence of irrelevant variables and noise, it is essential to carry out variable selection methods to pick the most relevant variables responding to the target properties instead of the full length of spectra [21, 43].

The joint analyses of chemometric statistics and variable selection algorithms has recently been used to eliminate the irrelevant variables and improve the model accuracy [9, 47]. The most common methods of variable selection are Genetic algorithm (Ga) [91], Regularized elimination procedure (Rep) algorithm [49], Iterative predictor weighting (Ipw) [22] and significant Multivariate Correlation (sMC) algorithm [79]. However, the comparison of variable selection algorithms along with PLSR for the prediction of multiple leaf nutrition traits is less studied.

*Sassafras tzumu* is a deciduous tree species that has colourful leaves in autumn. Zhejiang province in China is vigorously promoting the cultivation of colourful species making *S. tzumu* a famous tree species. It has been widely planted in Zhejiang province to develop the urban and mountain landscape [35].

Therefore, the aims of this research are to (1) test the capacity of reflectance spectroscopy to characterize the NBI, ANTH and FLAV with PLSR model; (2) find out the most optimal pre-processing method for these three leaf traits. (3) Identify the most important wavelength that related to NBI, ANTH and FLAV by four variable selection methods, including significant multivariate correlation (sMC), regularized variable elimination procedure (Rep), iterative predictor weighting (Ipw), and Genetic algorithm (Ga) variable selection; (4) estimate genetic parameters and correlations of NBI, ANTH and FLAV in *S. tzumu*.

## Methods and materials

### Materials

50 half-sib families of *S. tzumu* were selected for our study from 6 different regions. Trees were planted in 2016 using a randomised complete block by a 2 m × 3 m spacing in Changle Forest Farm Nursery (30° 27′ N, 119° 48′ E), Hangzhou, Zhejiang, China. Each family replicated 30 times with 5 replications and 6 individual trees per replication. In total, 1500 trees were planted.

### NIR spectra collection

Samples spectra data was collected through 5–6 leaves of each tree from the top to bottom with similar color on the same side in October 2018. The NIR spectra data was taken from the upside surface of the leaves three times with a handheld fibre optic contact probe from a field-based spectrometer (LF-2500, Spectral evolution, USA). Each spectrum took on average 32 scans with a range of 1100 to 2500 nm by a 6 nm resolution. All spectra were obtained from the leaves of 1500 trees, 500 trees leaves from these 1500 trees were sampled and placed in a marked paper bag and transferred to the refrigerator immediately for chemical measurement.

### Leaf FLAV measurement

Each leaf was ground into powder and being mixed with methanol for 24 h. 0.5 ml (1 mg/ml) extract of each sample was taken to mixed with methanol (1.5 ml), 10% aluminium chloride (0.1 ml), 1 M potassium acetate (0.1 ml) and distilled water (2.8 ml). The mixture was being placed under room temperature for 30 min and then measured at 415 nm for the absorbance by UV–Visible spectrophotometer (UV-1280, Shimadzu, Japan). The flavonoid content of the sample was accessed by the value of absorbance density [18].

### Pigment extraction and NBI estimation

A weighed circular piece cutting from each leaf was place into a mortar by a pestle ground with 100% methanol until the colour changed into white. The extract was being centrifuged for 6 min by 14,000 rpm at 4 °C and subsequently assayed by a UV–Visible spectrophotometer (UV-1280, Shimadzu, Japan). It conducted the equation and specific absorption in the wavelength which was reported by Wellburn [83]. The solution was mixed with 3 ml acidified methanol (1% HCl) at 4 °C with moderate shaking for 12 h and then being centrifuged for 10 min at 14,000 rpm. The extraction was then placed into the spectrophotometer, it took the absorption at 530 and 657 nm wavelengths to determine the ANTH concentration [76]. The NBI index was figured as the ratio of chlorophyll to flavonoid content.

### Model calibration and validation

The original five different types of pre-processing spectra (SNV, 1st, 2nd derivatives, SNV + 1st derivatives, SNV + 2nd derivatives) combined with PLSR [86] algorithm were compared in our study. The Savitzky–Golay smoothing [64] with a window size of 15 data points was applied in both 1st and 2nd derivatives spectra. PLSR models were generated with leave-one-out cross-validation for the prediction of ANTH, NBI, and FLAV content. Data were randomly split 200 times into calibration (80%) for model building and validation (20%) for model test respectively. Therefore, the PLSR model has been conducted 200 times for the evaluation of model performance. Each model combined with four variable selections (sMC, Ipw, Rep and Ga) was conducted to find out the most important spectral variables. The coefficient of determination (R^2^) and root-mean-square error (RMSE) in each model derived from both calibration (Cal) and validation (Val) were applied for the evaluation model performance.

### Statistical analysis

The estimation of genetic parameters were measured by a multivariate restricted maximum likelihood (REML) linear mixed model, details can be found in Li et al. [40]. The narrow sense heritability $({h}^{2})$ of trait $i$ and genetic correlations $( {r}_{{g}_{ij}})$ and phenotypic correlation ${( r}_{{p}_{ij}})$ between trait $i$ and trait $j$ were calculated as:

$$h_{i}^{2} = \frac{{2.5\sigma_{{f_{i} }}^{2} }}{{\sigma_{{f_{i} }}^{2} + \sigma_{{e_{i} }}^{2} }}$$

$$r_{{g_{ij} = }} \frac{{\sigma_{fifj} }}{{\sqrt {\sigma_{{f_{i} { }}}^{2} + \sigma_{{f_{j} { }}}^{2} } }}$$

$$r_{{p_{ij} = }} \frac{{\sigma_{fifj} + \sigma_{eiej} }}{{\sqrt {\left( {\sigma_{{f_{i} { }}}^{2} + \sigma_{{e_{i} { }}}^{2} } \right)\left( {\sigma_{{f_{j} { }}}^{2} + \sigma_{{e_{j} { }}}^{2} } \right)} }}$$

where ${\sigma }_{{f}_{i} }^{2}$ is the estimated family variance for trait $i$, and ${\sigma }_{{f}_{j} }^{2}$ is the estimated family variance for trait $j$,${\sigma }_{{e}_{i}}^{2}$ and ${\sigma }_{{e}_{j}}^{2}$ are the residual variances for trait $i$ and $j$, and ${\sigma }_{fifj}$ and ${\sigma }_{eiej}$ are the family and residual covariances between traits $i$ and trait $j$. The random effects of each family were set as breeding values. The realized genetic gain ($\Delta {G}_{R}$) was calculated by the difference between the mean breeding values of selected top ratio leaf traits and the total mean of the leaf traits.

R software (version 3.1.2) [66] was used for all of the data analysis. The *pls* package [50] in R was carried out for PLSR model building, and the *plsVarSel* [49] for variables selection, the *prospectr* package [75] for NIR spectra manipulation, the *lme4* package [4] for estimation of genetic parameters, and the *ggplot2* package [84] for visualization plot.

## Results

### Model performance

**Fig. 1:**
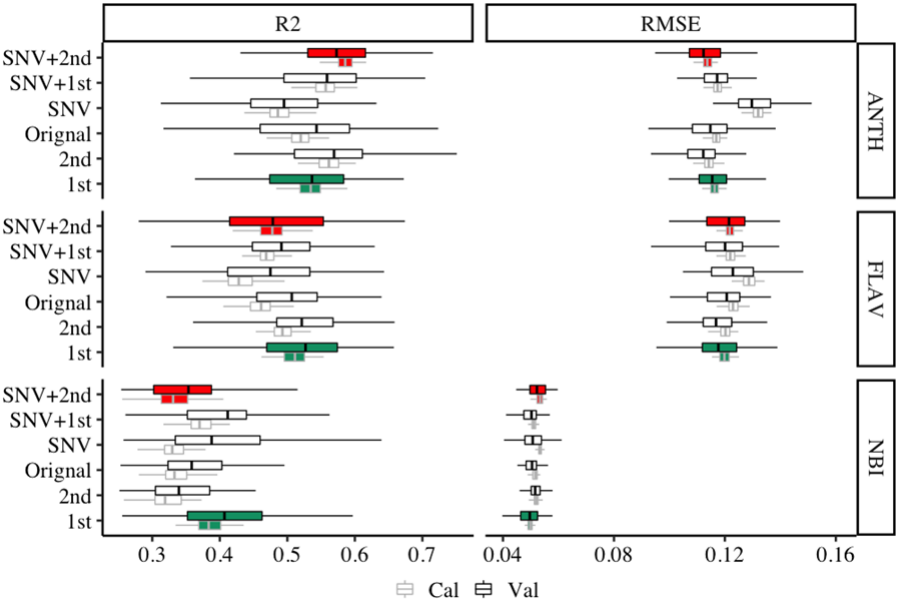
Distribution (95% confidence intervals) of calibration and validation statistics from 200 simulations of models predicting ANTH, FLAV and NBI with full length NIR spectra. Each model permutation included 80% of the data for internal calibration and the remaining 20% for validation. R^2^: coefficient of determination of cross-validation; RMSE: root-mean-square error of cross-validation; The black vertical line in each box represents median value, the red colour box represents the SNV + 2nd model. The green colour box represents the 1st model

Figure 1 displays the NIR spectral PLSR model for ANTH, FLAV and NBI traits. ANTH model has the highest accuracy, followed by FLAV and NBI model. The average of R^2^ and RMSE for these three models in calibration (Cal) sets are 0.54 (range: 0.43–0.63), 0.47 (range: 0.35–0.58) and 0.36 (range: 0.26–0.45), in validation (Val) sets are 0.54 (range: 0.28–0.75), 0.47 (range: 0.28–0.69) and 0.38 (range: 0.25–0.64) respectively. As for all spectral pre-processing models, SNV + 2nd derivative prediction model is found to be the highest well-performing for predicting ANTH concentration than the other pre-processing methods, with a mean R^2^_Cal_ and RMSE_Cal_ of 0.59 (range: 0.55–0.63), 0.11% (range: 0.11–0.12%), a mean R^2^_Val_ and RMSE_Val_ of 0.57 (range: 0.38–0.72), 0.11% (range: 0.09–0.13%), followed by 2nd, SNV + 1st, 1st, original with the mean of R^2^ in Cal is 0.56 (range: 0.42–0.75), 0.56 (range: 0.51–0.60), 0.53 (range: 0.48–0.59), 0.52 (range: 0.47–0.56), and RMSE 0.11% (range: 0.11–0.12%), 0.11% (range: 0.11–0.12%), 0.11% (range: 0.11–0.12%), 0.12% (range: 0.11–0.12), and in Val is 0.57 (range: 0.42–0.75), 0.54 (range: 0.30–0.70), 0.53 (range: 0.36–0.67), 0.52 (range: 0.32–0.72), and RMSE 0.11% (range: 0.09–0.13%), 0.12% (range: 0.10–0.14%), 0.12% (range: 0.10–0.14%), 0.11% (range: 0.09–0.14%) respectively. SNV shows the worst effect with the mean of R^2^ and RMSE for Cal and Val 0.49 (range: 0.44–0.54), 0.49 (range: 0.28–0.63), and 0.13% (range: 0.13–0.14%), 0.13% (range: 0.12–0.16%) respectively. However, 1st yields the best PLSR model in the prediction of FLAV and NBI than the other pre-processing model, with high mean R^2^_Cal_ R^2^_Val_ of 0.51 (range: 0.46–0.58), 0.52 (range: 0.29–0.68), and low mean of RMSE_Cal_, RMSE_Val_ of 0.12% (range: 0.11–0.13%), 0.12 (range: 0.10–0.12%) in FLAV model and high mean R^2^_Cal,_ R^2^_Val_ of 0.39 (range: 0.33–0.45), 0.41 (range: 0.26–0.60), and low mean of RMSE_Cal_, RMSE_Val_ of 0.05% (range: 0.05–0.05%), 0.05 (range: 0.04–0.06%) in NBI model respectively. The effect of SNV shows a poor prediction in the FLAV and NBI as well. The mean of R^2^_Val_ is 0.40 (range: 0.26–0.64) and 0.47 (range: 0.29–0.64) respectively.

**Fig. 2:**
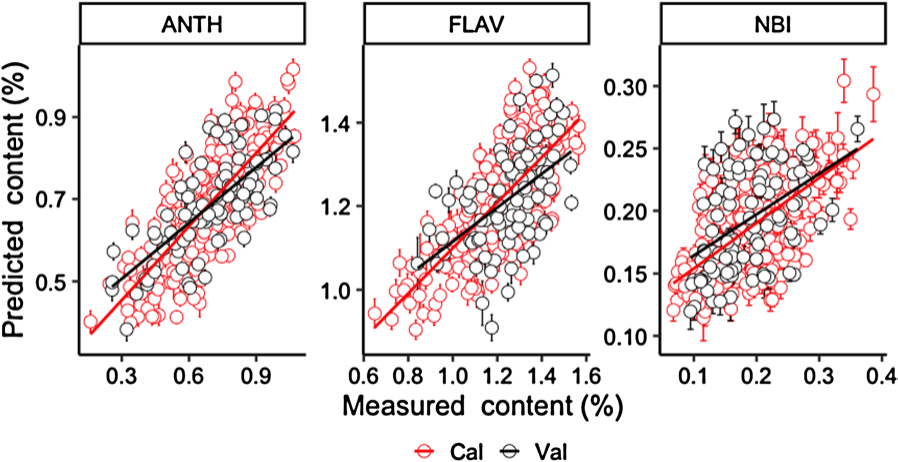
Measured and predicted ANTH, FLAV and NBI contents with full length of NIR spectra. Error bars for predicted values represent the standard deviations obtained from the 200 simulated models

The relationship between the predicted and measured content of Cal and Val datasets by ANTH model with SNV + 2nd derivative spectra, FLAV and NBI model with 1st derivative spectra were plotted in Fig. 2. The error bar represents the prediction error of 200 times per sample. It shows that due to the high accuracy of the ANTH and FLAV models, the predicted values are more correlated with the measured values, while the relationship between predicted and measured values of NBI model is relatively poor. Although the prediction accuracy of each model is different, the prediction error of the Cal and Val data sets is still small.

**Fig. 3:**
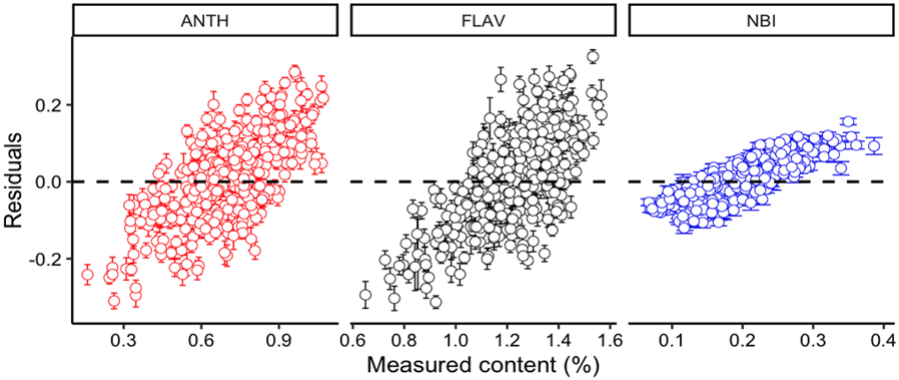
Residuals plotted against measured ANTH, FLAV and NBI with full length of spectra. Error bars for predicted values represent the standard deviations obtained from the 200 simulated models

The residual of the best processing spectra model for each leaf trait shows that all of these three models tend to be underpredicted when the measurement value is small. With the rise of the measurement value, the prediction value has the tendency of overprediction. The residual value of ANTH, FLAV and NBI model is between an acceptable range from − 0.3 to 0.3 (Fig. 3).

### Variable selection and model optimization

**Fig. 4:**
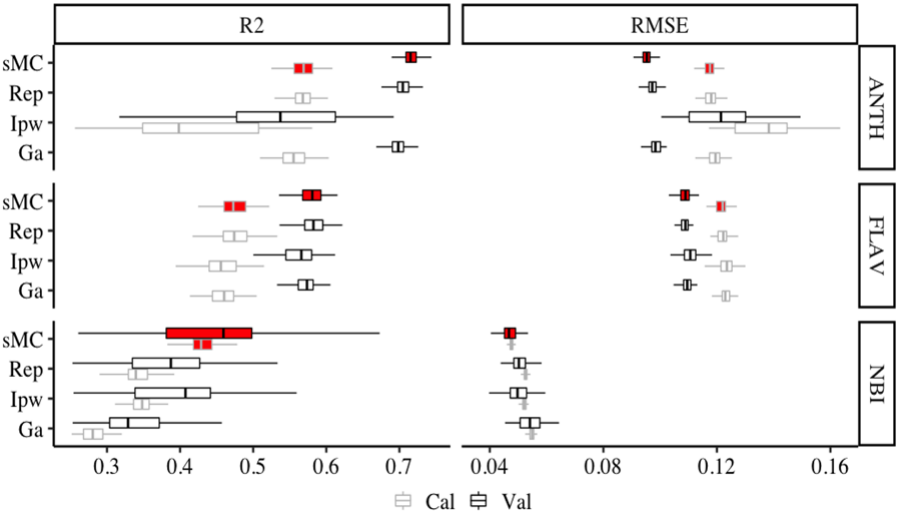
Distribution (95% confidence intervals) of calibration and validation statistics from 200 simulations for models predicting ANTH, FLAV and NBI contents using sMC, Rep, Ipw and Ga variable selection. Each model permutation included 80% of the data for calibration and the remaining 20% for validation. R^2^: coefficient of determination of cross-validation; RMSE: root-mean-square error of cross-validation; The black vertical line in each box represents median value, the red colour box represents the sMC model

**Fig. 5:**
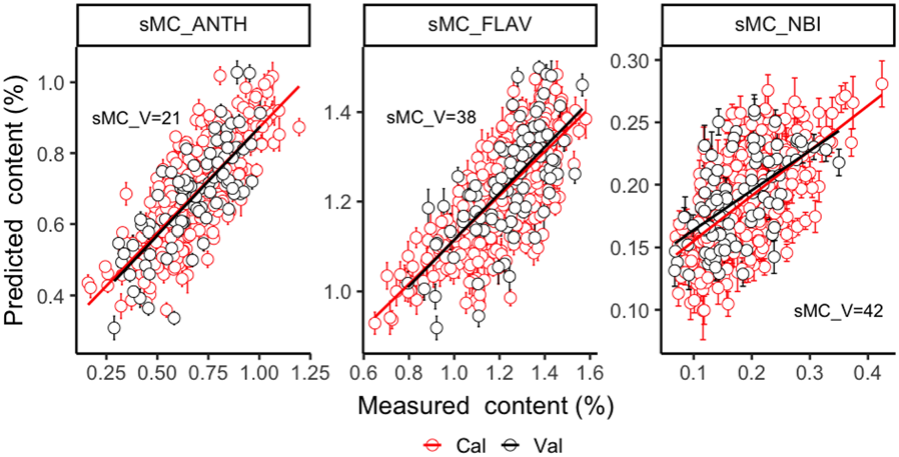
Measured and predicted ANTH, FLAV and NBI contents with sMC selected NIR spectra. Error bars for predicted values represent the standard deviations obtained from the 200 simulated models. sMC_V: the total selected number of variables

**Fig. 6:**
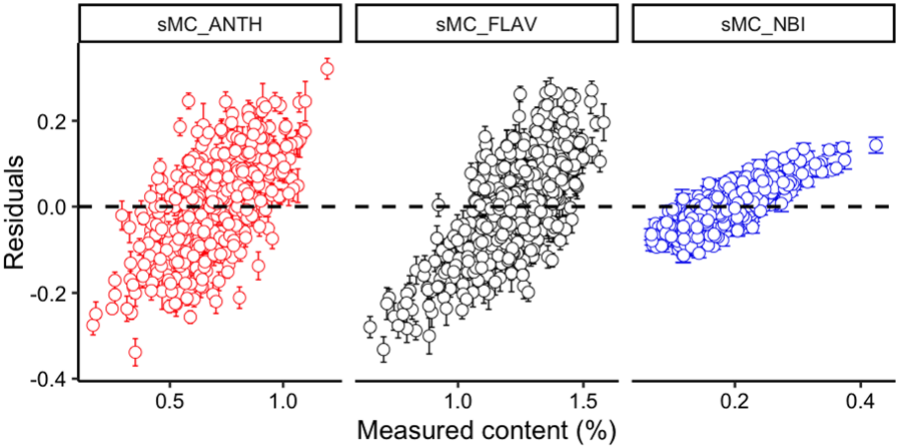
Residuals plotted against measured ANTH, FLAV and NBI with sMC selected spectra. Error bars for predicted values represent the standard deviations obtained from the 200 simulated models

Four types of variable selection methods were compared to test the performance of ANTH, FLAV, and NBI PLSR models (Fig. 4). The prediction accuracy of ANTH, FLAV, and NBI PLSR models was enhanced much better than the full-length spectra models by these four different variable selection methods. ANTH model still holds the highest R^2^ and RMSE value in both Cal and Val data, followed by the FLAV and NBI model. The highest prediction model for ANTH, FLAV and NBI was found through sMC-selected NIR spectra variables with the mean R^2^_Val_ of 0.72 (ranged: 0.69 to 0.75), 0.58 (ranged from: 0.54 to 0.62), 0.44 (ranged from: 0.26 to 0.67), and the mean RMSE_Val_ of 0.10% (range: 0.09–0.10%), 0.11% (range: 0.10–0.12%), 0.04% (range: 0.04–0.05%) respectively. The sMC_PLSR models reached a more stable prediction with less than 16% of full length of spectra on each leaf trait (Fig. 5), and having a similar residual range to the model with full length of spectra (Figure 6).

**Fig. 7:**
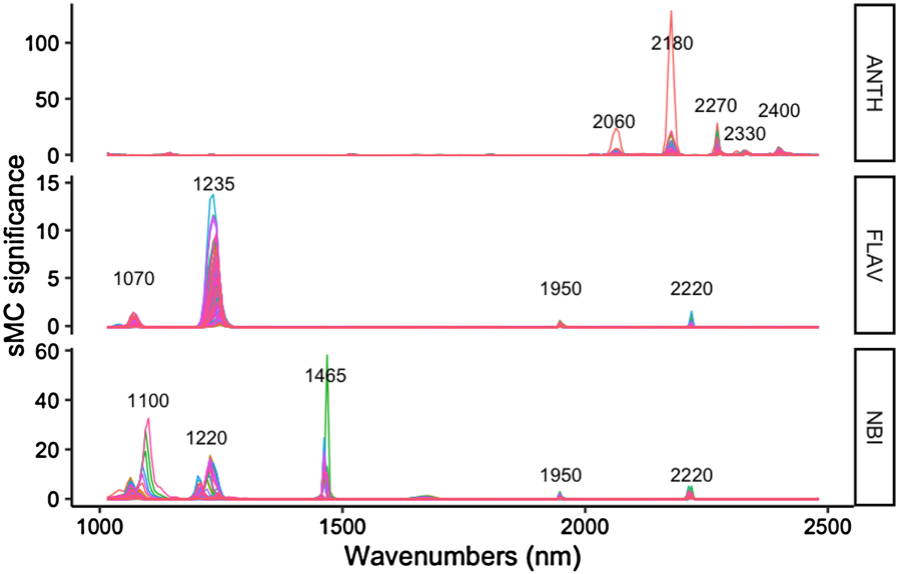
Spectra influence in ANTH, FLAV and NBI models that randomly being conducted 200 times; each line means one time of modelling with sMC variable selection

Figure 7 displays the important variable information area selected by sMC variable selection method in the ANTH, FLAV and NBI model which conducted 200 times on each model. Even the predicted model of three leaf traits was being run 200 times, sMC variable selection brought out stability for the selected important variable areas with a few relative spectral regions in prediction models. The variables at 2060, 2180, 2270, 2330 and 2440 nm are considered as the vital roles in the construction of ANTH prediction model. As for FLAV, 1070, 1235, 1950 and 2220 nm are the most important areas. Spectroscopic variables at 1100, 1220, 1465, 1950 and 2220 nm make a critical difference in the NBI predictive model.

### Heritability, genetic and phenotypic correlation among traits

Table 1 shows the correlation (genetic and phenotypic) and heritability of three traits. Leaf ANTH produces the highest heritability of 0.78, followed by FLAV and NBI with *h*^*2*^ of 0.58 and 0.61 respectively. There has no significant genetic and phenotypic correlation between ANTH, FLAV and NBI. FLAV was found to have the highest positive genetic correlation with ANTH with a value of 0.36.

**Table 1:** The heritability, genetic (above diagonal (italic)) and phenotypic correlation (below diagonal) between ANTH, FLAV and NBI traits with the standard error between parentheses

Traits	ANTH	FLAV	NBI	*h*^2^
ANTH		*0.36* (*0.01*)	*0.11* (*0.02*)	0.78 (0.10)
FLAV	0.16 (0.03)		*0.09* (*0.01*)	0.58 (0.11)
NBI	0.09 (0.01)	0. 12 (0.01)		0.61 (0.08)

### Family selection

**Fig. 8:**
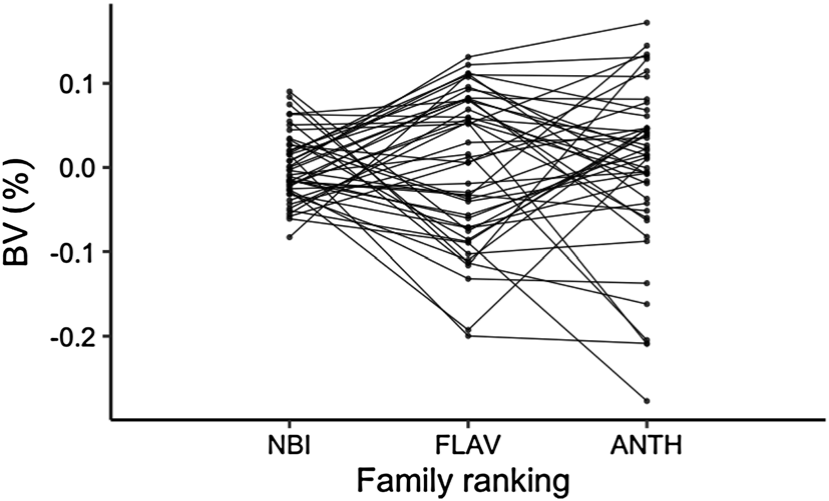
Family ranking for ANTH, FLAV and NBI content in *Sassafras tzumu* at age 2. Family values are expressed as deviation from each trait mean. BV: Breeding values

The best models of ANTH, FLAV and NBI were applied to predict the remaining 1000 trees spectra. In total, 1500 trees of 50 families were selected for breeding analysis. Figure 8 shows the distribution of three leaf traits in the ranking of breeding value from 50 families. The ranking of three leaf traits in different families is inconsistent as well as a part of families consistently displaying in the breeding value, which explains that it is feasible to make a family selection of ANTH, FLAV and NBI at the same time through genetic selections.

**Fig. 9:**
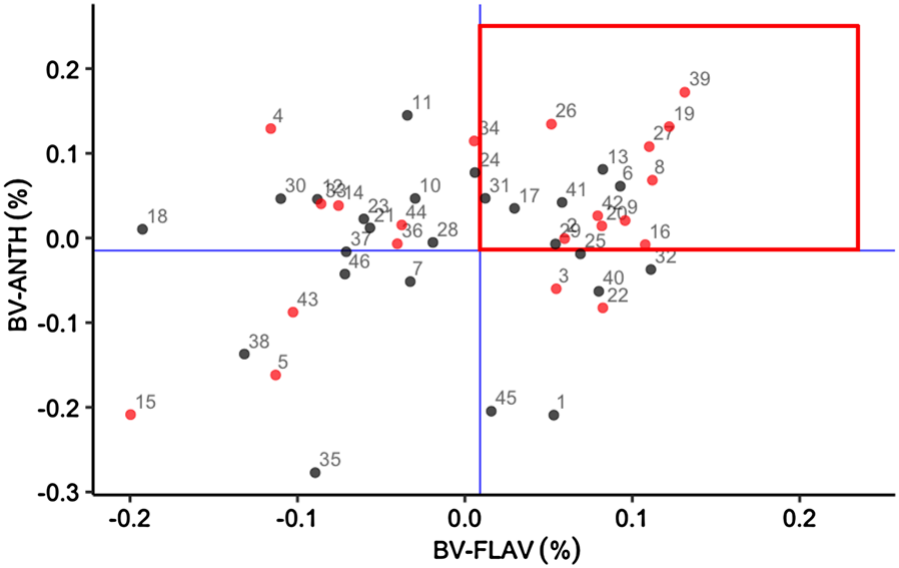
Relationship between ANTH, FLAV and NBI content breeding values of *Sassafras tzumu* families at age 2. BV-ANTH: breeding value of ANTH; BV-FLAV: breeding value of FLAV; the blue solid line: the mean value of each trait breeding value; red square: the region that most interesting. The number of each dot: family number

Figure 9 demonstrates the breeding value distribution of 50 families of three leaf traits. The blue solid lines represent the average of ANTH and FLAV respectively. The families with a higher NBI breeding value than its mean are shown in red, and below the mean are in black. There are 16 families have high FLAV and ANTH breeding value, 10 families with a high breeding value will be selected If NBI breeding values are required to be above mean. These families can be further taken as genetic family materials for second-generation breeding.

## Discussion

The health of tree growth is dictated by main factors, such as soil, nutrients, environment, genetic and so on. N is a key role of nutrient which highly influences the tree growth.

The internal N cycling in trees [78] is a hot topic in numerous studies [24, 38, 53]. However, the measurement of N concentration limits the access to the further study. In this study, the field-base reflectance spectroscopy has been proved to be a useful method to characterize the plant nutrition properties in fresh leaves. The SNV + 2nd derivative spectra for ANTH, and 1st derivative spectra for FLAV and NBI have been identified to increase the model accuracy when calibrating the PLSR prediction models. Incorporate with spectra variable selection, the model accuracy has been significantly improved with less variables for the prediction of leaf nutrition traits. Our model offered a reliable result for predicting the FLAV content in fresh leaf (R^2^_Val_ = 0.58, ranged from: 0.54 to 0.62), which was lower than the result reported for *fresh Ginkgo biloba* leaf in different colors (R^2^_CAL_ = 0.82 and RMSE = 2.62%) [71]. The variability lessened by small range of NBI value lead to an inefficient prediction [5].

Conversely, our prediction of ANTH content result illustrates a suitable accuracy than the other two leaf traits, with a mean R^2^_Val_ of 0.72 (range: 0.69–0.75) and a mean of RMSE_Val_ of 0.09% (range: 0.09–0.10%). Similar result was discovered in wine grapes by NIR hyperspectral imaging and PLSR model, which gave R^2^ of 0.84 and RMSEP of 0.013% for estimating ANTH content.

A robust statistical methodology for model calibration, which was first conducted by Couture et al. [12] to predict plant leaf secondary metabolites with reflectance spectroscopy, was applied in our study. It has being run 200 randomized simulations for calibrating the models to provide an estimation of the model uncertainty and overall stability (Figs. 1, 2, 3, 4, 5, 6, 7). It is similar to our previous study which takes use of filed spectroscopy to predict the leaf colour and chlorophyll content [41]. Random sampling [65] and Kennard-Stone sampling algorithm [42] in other studies, which sample only once for model calibration, may cause instability for model prediction. Thus, we highly recommend using this methodology for model calibration and validation on NIR analysis.

The NIR spectra involves not only the favourable information but noise and irrelevant information which will encumbrance the accuracy of prediction model. Therefore, variable selection is regarded as an efficient way to find out the most important wavelengths which contributes the minimum error for model calibration and helps to reduce the model processing time for spectral models. Variables in the spectrum play a key role in the predictive accuracy of the model. The spectral information is extensive along with the relevant and irrelevant information, both of which will overlap to interfere the model construction of the useful information and the PLSR model with a specific trait [88]. Thus, it is vital to screen important variables for spectral information. In this study, four variable selection methods were compared to pick the best variable selection method. It shows that the sMC-PLSR model efficiently identified the key wavelengths and enables us to select a small set of variables to yield a promising and robust calibrated model for the prediction of ANTH, FLAV and NBI. Our results support the research announced by Li and Altaner [39], who successfully took the sMC variable selection method to improve the accuracy of an NIR calibration model on the prediction of extractives contents in heartwood of *Eucalyptus bosistoana* trees, and Li et al. [41] who found that sMC selection algorithm held the advantage of finding the most relevant variables for the prediction of leaf chlorophyll content and colour parameters. Some studies also states that significance multivariate correlation (sMC) [79] is a positive algorithm to remove confounding effects from NIR calibrations [85].

Several important variables which are related to the ANTH, FLAV, and NBI have been selected similarly in each model, including the range at 2060, 2180, 2270, 2330 and 2440 nm for ANTH, 1070, 1235, 1950, 2220 nm for FLAV, and 1100, 1220, 1465, 1950, 2220 nm for NBI respectively. As reported by Ramirez et al. [67], the regions around 2060, 2180, 2270, 2330 and 2440 nm are mostly associated with O–H and C–H stretching vibrations as well as the starch and sugar [17]. However, in our study, these regions have been ignored. The regions around 1070, 1100, 1220, 1235 nm are mainly assigned to the 1st overtones of C–H combination bands and 1st and 2nd overtones of O–H and N–H stretching vibrations, while the bands around 1465 nm are mostly related to the 1st overtones of O–H stretching vibration, both of which are associated with starch and protein [14, 16, 37]. In NIR spectra, water has a wide absorbance region which is a major influence on the other chemical information because of spectra overlap. In our study, the band around 1950 nm related to the water has less contribution to the FLAV and NBI model and no influence on the ANTH model. It probably influences the accuracy of model for the prediction of FLAV and NBI. Correlational study was found by Min et al*.* [55], who stressed that the regions of 1910 and 1938 nm highly related to water might have a strong impact on the N concentration prediction.

Trees N internal cycling is considered as one of the major ecology factors for tree growth and is an augment for the tree uptake of soil N [51]. In addition, it also helps to understand numerous aspects of plant ecology, for instance, to evaluate the effect of the N storage and remobilization in different part tissues of trees in relation to current demands for growth [70], to find out the role of N on growth stress, the relationship with N deposition in forest [28, 29] and the relationship with dynamics of carbon recourse in trees [30, 82]. Our fast and accurate measurement of N index, including ANTH, FLAV and NBI traits of trees with NIR spectroscopy provides an advanced way for the study of N internal cycling and allows to quickly measure large number of samples.

In this study, we continue to use the coefficients of 1/2.5 for the calculation of heritability of ANTH, FLAV and NBI traits based on our previous study to avoid the assembling of half-siblings and inbreeding effects [41]. The moderate heritability of ANTH, FLAV and NBI was found, with the value of *h*^*2*^ ranging from 0.61 to 0.78. The leaf ANTH heritability of 0.78 in our study is similar to the result found by Yihu et al. [77] who figured out the anthocyanin content heritability ranging from 0.79 to 0.91 in leaves of chili pepper higher than 0.29 reported in the leaf of Aspen (*Populus tremula* L.) [69]. For FLAV, a significant high rang of heritability from 0.94 to 0.99 was reported in the leave of Ginkgo Trees [90] which was much higher than our study (*h*^*2*^ = 0.58). It indicates that genetic control capacity is different between species even the same traits. Our study proved that there is also a potential for the selection of NBI traits in breeding programs even with less study on the estimation of NBI heritability.

The consistence of families ranking of ANTH, FLAV and NBI indicates that the selection for a good leaf nutrition tree is workable, and the selection of qualified nutrition plant is supposed to involve multiple traits, which will afford a stable inheritance.

## Conclusion

In conclusion, NIR spectroscopy is potentially taken to estimate the nutrition related traits by fresh leaf. With the small prediction error, the tree breeding programs can be successfully achieved based on the relative prediction value. Our study provides an alternative way for the N index traits and open a door to the efficient analysis of the internal N cycling in trees. The pre-processing method and variable selection highly influence the performance of model prediction. Our study found that by using of 1st and SNV + 2nd derivative spectra processing method and sMC variable selection algorithm, the PLSR models have been highly improved. In addition, the repeated spectral statistical methodology that we applied provided an efficient way to deal with variation in calibration data and generate information on the response of plant nutrition traits with NIR spectra. NIR model serves as an efficient tool for the estimation of genetic parameters and breeding selection in high throughput way to improve the leaf traits quality.

## Data Availability

Not applicable.
